# Exploring the Intersection Between Seizures, Traumatic Brain Injury, and the Risk of Incident Cognitive Impairment in Cognitively Normal Older Adults

**DOI:** 10.1002/alz.092832

**Published:** 2025-01-03

**Authors:** Ganesh M. Babulal, Jean‐Francois Trani, Yiqi Zhu

**Affiliations:** ^1^ Washington University School of Medicine, St. Louis, MO USA; ^2^ Natiobal conservatory of Arts and Crafts, Paris, NA France; ^3^ Adelphi University, Garden City, NY USA

## Abstract

**Background:**

Seizures, traumatic brain injury (TBI), and dementia increase in prevalence with age. However, the role of seizure and TBI on cognitive impairment risk (CI) is unclear. This study investigated how a diagnosis of seizures and TBI was associated with the progression to CI and assessed the role of medications.

**Method:**

We analyzed data from the National Alzheimer’s Coordinating Center (NACC) (June 2020 data‐freeze). Inclusion criteria required participants 1) aged 65 years or older at their first visit, 2) cognitively normal at baseline (Clinical Dementia Rating [CDR] score of 0), and 3) complete information on race, age, gender, and education. 8,139 participants were included and followed until dropout or the first instance of CI progressing CDR 0 to 0.5. Seizure and TBI were determined by the clinician’s judgment based on medical history and the participant’s annual visits. Medication categories were derived by NACC using Ultum/Lexi‐Comp© therapeutic drug categories. Age, sex, race/ethnicity, and education were included as control variables. Cox proportional hazards model examined the associations between the diagnosis of seizure, TBI, or both and the progression of cognitive impairment measured by CDR. To account for selection bias, we applied propensity score weighting (PSW) with the inverse probability treatment weighting (IPSW) technique.

**Result:**

Unadjusted survival models found a diagnosis of seizures significantly increased the hazard ratio (HR) of CI progression by 58% (p < 0.05), while the diagnosis of TBI increased the HR by 28% (p < 0.05). After applying IPSW to reduce the selection biases, the diagnosis of seizures increased the HR for CI by 39% (p < 0.05), TBI increased it by 25% (p < 0.001), and having both seizure and TBI diagnoses increased the risk of CI by 56% (p < 0.05). The use of antipsychotic, anxiolytic, sedative, or hypnotic agents and angiotensin‐converting enzyme (ACE) inhibitors was found to reduce the risk of CI significantly.

**Conclusion:**

The presence of seizure or TBI was significantly associated with the increased risks of CI. Antipsychotic, anxiolytic, sedative, or hypnotic agents and angiotensin‐converting enzyme (ACE) inhibitors were associated with a reduced risk, emphasizing the importance of early treatment.

